# Some Problems Which Press

**Published:** 1915-10-30

**Authors:** 


### NATIONAL INSURANCE ACT DEVELOPMENTS.

### SOME PROBLEMS WHICH PRESS.

A good deal has been spoken and written during the past few weeks on the subject of the amendment of the National Insurance Acts. It has been averred in certain quarters, for instance, that the abolition of the four separate Commissions is imminent, and that the rights and privileges of the self-governing approved societies which at present administer the Acts are to be curtailed, even if the societies are not actually to be abolished in favour of direct Governmental control. There is, however, so far as we can ascertain, no substantial authority for any of these sweeping assertions, though the evasive answer given by the Prime Minister to a question in the House of Commons on September 29, when he said that he could make no statement on the subject of the amalgamation of the Commissions until he had received the report of the Retrenchment Committee, may have lent some colour to the suggestion. Another point which has not escaped attention is the fact that recent vacancies which have occurred in the ranks of the Commissioners, through death, resignation, and promotion, have been left unfilled.

That National Insurance administration must be included with that of other Government departments in the purview of the Retrenchment Committee is, of course, a foregone conclusion, but it does not follow that the Committee will consider it wise policy at the present juncture to suggest any radical changes in the original plan upon which the system is based. Any fundamental alteration of the system would require a vast amount of consideration, both in principle and in detail—more than could, in fact, now be devoted to the subject either by Parliament or by the country generally. Furthermore, it is probable that in the initial stages, lasting perhaps for two or even three years, any general alteration in the methods of administration would entail increased rather than reduced expenditure; and, consequently, it is by no means certain that the present time is the most suitable for the introduction of any fundamental change, even if another plan were proved to be better than the one now in operation.

### The Valuation of Approved Societies.

We have now had rather more than three years' practical experience of the National Insurance Act, so far as the collection of contributions is concerned; and in January next the first three years of the payment of benefits will have been completed. In normal circumstances the first valuations, by which the financial results of the working can alone be satisfactorily tested, would be in hand. But, so far, no announcement has yet been made by the Insurance Commissioners as to when the valuations are to be undertaken, and it appears that the stocktaking—as the valuation really is—is to be indefinitely postponed. It has to be remembered in this connection that there was no binding obligation upon the Commissioners to require the valuation to be made at the end of the first three years, though it is clear that triennial valuations were contemplated by the framers of the Act. The Act of 1911 (section 36) provides that a valuation of the assets and liabilities of every approved society shall be made by an authorised valuer at the end of every three years dating from the commencement of the Act (July 15, 1912), or at such other times as the Insurance Commissioners may appoint. The times so appointed may, in fact, be at shorter or longer intervals, either regular or irregular, and may apply to all approved societies or to any particular societies as the Commissioners may determine. Thus it will be observed that the Commissioners have full statutory authority for deferring the date of the valuation beyond the time originally contemplated.

It must be remembered that the labour involved in making the valuations must necessarily be of a complicated and technical character, and will necessitate the employment not only of the skilled valuers with actuarial qualifications, but also of large numbers of clerks, all more or less familiar with National Insurance work; and at the present time such labour is difficult to obtain. This is one side of the question, but the other side must not be forgotten.

### Why the Valuations should be Made.

The object of the valuations is not merely academic, nor to furnish evidence of success for the gratification either of those who inaugurated the scheme or of those who have taken a share in carrying it out. The object is rather to guard against the possibility of societies, which have been given Government approval for the purpose of national health insurance, falling into a state of insolvency, and to provide the means whereby, if deficiencies are shown, the matter may be rectified before it becomes too late to take effective action. This aspect of the question must be borne in mind all the more carefully when it is remembered that national health insurance is a new venture, and that the relation between benefits and payments was based upon estimates which had to be made from data not suitable in all respects for the purpose. It has, in fact, been proved, without the more extensive analysis required by a formal valuation, that the original estimates, so far as the sickness experience of women's societies as a whole is concerned, were deficient, and it is to guard against the deficiencies attaining unmanageable proportions that the valuations should be made at the earliest possible moment.

The decision of the Insurance Commissioners between these rival forces—the unsettled and abnormal conditions and the consequent lack of labour on the one hand, and the necessity for preventing the whole scheme falling into insolvency on the other—is no light task. It is hardly surprising in the circumstances that there should be sharp differences of opinion on the subject. Approved society officials, already harassed and overburdened with the administrative work, with difficulties in the way of obtaining efficient help for normal duties, are not anxious to add to their immediate burden. On the other hand, such impartial and detached observers as, for instance, Mr. Handel Booth are naturally pressing that the valuation should be made, or that a thorough inquiry should be instituted at once into the entire scheme.

Having thus stated the broad outlines of the present uncertainty as to the future of National Insurance, the matter must be left in the hands of the responsible officials; but it must not be forgotten that National Insurance does not exist for the benefit of the Commissioners, but the Commissioners for the well-being of National Insurance. National Insurance, furthermore, stands, or should stand, for the well-being in matters of health of the industrial population, and for this reason it is necessary to assert that in spite of all present difficulties occasioned by the war, or even, perhaps, because of those difficulties, it is imperative that public attention should not be allowed to be entirely diverted from the National Insurance problems.

Suggested Amendments.

In order to prepare for eventualities, various suggestions have recently been put forward for consideration in connection with the possible amendment of the Acts. One of the most useful summaries of these suggestions that we have seen is given in the *National Insurance Gazette* for October 23. The list is described as "Friendly Societies' Proposals," and, among others, contains the following:—

(1) That the different classes of contributors should be done away with, and that there should be only two classes—men and women.

(2) That the varying rates of contribution (low wage, etc.) should be abolished, and one rate for all men and one for all women introduced.

(3) That there should be one rate of benefit applicable to all male contributors and one rate applicable to all females.

(4) That the regulations as to arrears should be simplified.

(5) That in place of the options at present given to female contributors on marriage, a portion of the contributions paid should be refunded.

(6) That (*a*) the sickness benefit of members without dependants in sanatoria should be placed on the same footing as that of similar members in hospitals; (*b*) that benefit of members who die in hospitals should be paid to their relatives; (*c*) the question of payments to dependants should be placed on a more definite basis.

And (7) that the position of sailors and soldiers be simplified.

The present complicated classes of insured persons, entitled to varying rates of benefit, and chargeable with varying rates of contribution, entail an almost endless amount of labour which the extra degree of equity as between the classes hardly appears to justify. There are individual cases of hardship even under the present complicated scheme, and it is open to question whether these hardships would be materially increased if the same arrangements were made applicable to all, while it is certain that the whole body of insured persons would gain by the saving of administrative expenditure, and many individuals at present employed in administering the Acts would be set free for more productive work.

